# A Why Open Testicular Mapping (OTEM) Should Precede, and Often Replace, Micro-TESE in Nonobstructive Azoospermia

**DOI:** 10.1590/S1677-5538.IBJU.2025.0582

**Published:** 2026-03-16

**Authors:** Felipe Placco Araujo Glina, Marcelo Vieira, Eduardo Mazucato, Sidney Glina

**Affiliations:** 1 Centro Universitário FMABC Disciplina de Urologia Sandro André SP Brasil Disciplina de Urologia do Centro Universitário FMABC, Sandro André, SP. Brasil; 2 Projeto Alfa São Paulo SP Brasil Projeto Alfa, São Paulo, SP, Brasil

**Keywords:** Non-obstructive azoospermia, testicular sperm extraction, micro-TESE, open testicular mapping, male infertility, sperm retrieval

## COMMENT

Non-obstructive azoospermia (NOA) represents the most severe form of male infertility, poses significant challenges for clinical management. For men with NOA, the only opportunity for biological fatherhood depends on retrieving testicular spermatozoa to be used in intracytoplasmic sperm injection (ICSI). Currently, the gold-standard technique for this purpose is microdissection testicular sperm extraction (micro-TESE), a procedure first described by Schlegel in 1999 (1).

Micro-TESE, a microsurgical inspection of the testicular parenchyma under an operating microscope, allows identification of seminiferous tubules with focal spermatogenesis in approximately 40% to 60% of cases (1, 2).

However, micro-TESE has several drawbacks. The procedure is costly, as it requires both a surgical microscope and a highly trained microsurgical team. The cost and limited availability of operating microscopes, still lacking in many centers, have led many urologists to continue performing conventional TESE (2) or to adopt alternative methods such as loupe-assisted microdissection (I-TESE) (3), despite their inferior outcomes relative to micro-TESE. Moreover, hormonal alterations following micro-TESE have also been reported, with studies describing a transient decline in serum testosterone levels from 303 ng/dL to 248 ng/dL. Testosterone recovery to baseline may take up to 18 months in 95% of patients, and a small subset of patients may develop persistent hypogonadism (4).

In this context, open testicular mapping (OTEM), first described by Vieira et al. (5), has emerged as a less invasive and cost-effective alternative. The technique involves exposure of the testicle through a scrotal incision, followed by perforation of the tunica albuginea with a large-bore (19-gauge) needle. Manual compression of the testicle allows extrusion of testicular parenchyma through the puncture, which is then gently collected with microsurgical forceps. The number of biopsies, usually ranging from 12 to 16 depending on testicular volume, is distributed across the entire testis to ensure comprehensive sampling of the parenchyma. When immediate evaluation by an embryologist is available at the fertility laboratory, the procedure can be discontinued as soon as spermatozoa are identified in one of the earlier samples. The puncture sites in the albuginea do not require suturing. In their original study, Vieira et al. reported a sperm retrieval rate of 54% in 92 men with histologically confirmed NOA (5).

Subsequent studies have corroborated the effectiveness of OTEM. Lopes et al. evaluated 118 NOA patients who underwent this technique and reported a sperm retrieval rate of 55.8%. Among the 67 couples who proceeded to in vitro fertilization (IVF), fertilization, clinical pregnancy, and live birth rates were 62.1%, 46.3%, and 44.3%, respectively (6).

One of the pathophysiological explanations for OTEM's efficacy lies in the heterogeneous distribution of spermatogenesis within the testicular tissue of men with NOA. Jarvi et al. (7) performed fine-needle aspiration (FNA) mapping in 82 men with previously failed micro-TESE and found sperm in 29.3% of cases. Notably, the authors demonstrated that residual spermatogenesis was preferentially located in the peripheral rather than central regions of the testis. Because OTEM samples primarily the subcapsular region, this finding may help explain OTEM's success rate despite being a less invasive approach.

OTEM offers clear advantages: it is less expensive, does not require a surgical microscope, and, by avoiding a large albugineal incision, is less invasive and may reduced testicular morbidity. Importantly, a failed sperm retrieval with OTEM does not preclude proceeding with micro-TESE in the same operative session, offering a stepwise and cost-effective approach. In light of the above, we encourage and propose that urologists perform OTEM prior to micro-TESE in their next NOA case, as in approximately 55% of patients, micro-TESE may prove unnecessary.

**Table:** Comparison between Micro-TESE and OTEM.

Characteristic	Micro-TESE	OTEM
Invasiveness	High (large albugineal incision)	Low (multiple punctures)
Microscope required	Yes	No
Cost	High	Low
Risk of hypogonadism	5% (4)	Theoretically lower
Sperm retrieval rate	40–60% (1, 2)	~55% (5, 6)
Allows sequential procedure	Not applicable	Yes (micro-TESE may follow)

## Data Availability

All data generated or analysed during this study are
